# Auditory Processing of the Brain Is Enhanced by Parental Singing for Preterm Infants

**DOI:** 10.3389/fnins.2022.772008

**Published:** 2022-04-04

**Authors:** Eino Partanen, Gustaf Mårtensson, Pernilla Hugoson, Minna Huotilainen, Vineta Fellman, Ulrika Ådén

**Affiliations:** ^1^Cognitive Brain Research Unit, Department of Psychology and Logopedics, Faculty of Medicine, University of Helsinki, Helsinki, Finland; ^2^Center of Functionally Integrative Neuroscience (CFIN), Department of Clinical Medicine, Aarhus University, Aarhus, Denmark; ^3^Centre of Excellence in Music, Mind, Body and Brain (MMBB), Department of Psychology and Logopedics, Faculty of Medicine, University of Helsinki, Helsinki, Finland; ^4^Department of Women and Children’s Health, Karolinska Institute, Stockholm, Sweden; ^5^Department of Music, Art, and Culture Studies, Faculty of Humanities and Social Sciences, University of Jyväskylä, Jyväskylä, Finland; ^6^Department of Nursing Science, Sophiahemmet University, Stockholm, Sweden; ^7^CICERO Learning Network, Faculty of Educational Sciences, University of Helsinki, Helsinki, Finland; ^8^Department of Pediatrics and Clinical Sciences, Lund University, Lund, Sweden; ^9^Children’s Hospital, University of Helsinki, Helsinki, Finland

**Keywords:** auditory event related potential, auditory processing, infant-directed singing, mismatch response, preterm birth, sound discrimination

## Abstract

As the human auditory system is highly malleable in infancy, perinatal risk factors, such as preterm birth, may affect auditory development. In comparison to healthy full-term infants, preterm infants show abnormal auditory brain responses at term age, which may have long-term detrimental outcomes. To achieve an optimal neonatal care environment for preterm-born infants, many early interventions have been developed. Musical interventions developed for neonatal intensive care units (NICUs) have shown beneficial effects on vital functions and weight gain of preterm infants and might also influence basic auditory processing and thereby enhance outcomes. In the present study, we tested the effect of parental singing during kangaroo care on auditory processing of standardized audio stimuli. Preterm infants (born between 24 and 32 weeks of gestation) were randomized to singing intervention (*n* = 13) or control (*n* = 8) groups. The auditory processing was tested using two audio paradigms assessed with magnetoencephalography (MEG) at term corresponding age. To verify that the paradigms elicit responses in MEG, we studied 12 healthy full-term infants. In the singing intervention group, parents were instructed by a music therapist twice a week for 4 weeks to sing or hum during kangaroo care in an infant-directed way. The control group received standard kangaroo care. The results show that the infants in the singing intervention group show larger neural responses than those in the control group when controlling for the total amount of singing during kangaroo care. Our findings suggest that incorporating singing into kangaroo care may be beneficial for preterm infants, but the effect may not be due to exposure to singing but instead positive parenting, improved parental self-esteem and improved caregiver sensitivity.

## Introduction

Newborn infants are born with extensive knowledge of their auditory surroundings, which they use in social interactions with their caregivers and which forms a basis for developing language skills (Huotilainen, 2010). These early auditory skills are learned *in utero* (Partanen et al., 2013a). Since even a fetus can learn and react in noticeable ways, it allows many parents to interact with their fetus, for example by various kicking games, helping them to prepare for the birth of their child while reinforcing attachment formation after birth (Moon and Fifer, 2000). In preterm infants, this intrauterine development is disrupted, and may, in association with other perinatal risk factors, predispose to neurodevelopmental challenges.

Worldwide, 6–12% of all children are born preterm (World Health Organization, 2018), that is, born before 37 weeks of gestation (GW). While the majority of these children develop normally, infants born before 32 GW are at high risk for developmental delays (Anderson and Doyle, 2004). As the number of preterm births are increasing globally (Vogel et al., 2018) in parallel with decreasing mortality, especially for preterm infants at the greatest risk (Santhakumaran et al., 2018), the number of infants at risk of developmental challenges is increasing. Thus, identifying cost-effective ways to ameliorate potential neurodevelopmental deficits arising from preterm birth is important.

A commonly used method to improve well-being in preterm infants is kangaroo care, where the infant is placed in skin-to-skin contact with their caregiver. Kangaroo care has been shown to be very beneficial in many ways, for example by reducing mortality and infection rate while improving weight gain (Conde-Agudelo and Diaz-Rossello, 2016). However, from a neurodevelopmental viewpoint, kangaroo care may not yield tangible benefits (Conde-Agudelo and Diaz-Rossello, 2016). Indeed, many studies on the relation between kangaroo care and neurocognitive outcomes have had relatively short follow-up periods (2–18 months, Ohgi et al., 2002) and shown small effects. The handful of long-term follow-up studies have found only small benefits in, for example, executive functions (Feldman and Eidelman, 2003). Thus, while the benefits of kangaroo care are evident in the neonatal period, it may not provide support for those neurocognitive domains, such as language, that can have a large impact on infant development.

Indeed, studies suggest that language is one of the cognitive functions that may be delayed in preterm infants, even in absence of general cognitive delay (Pascal et al., 2018). This may predispose preterm children to poorer developmental trajectories. For example, at age 6 years, preterm-born children fared more poorly than full-term at tasks involving vocabulary, grammar, and phonological awareness, even when controlling for general cognitive capabilities and neonatal morbidities (Guarini et al., 2009). Poorer language outcomes in preterm infants were also seen in a large family study with over 25,000 siblings (Zambrana et al., 2020). Although preterm birth seems to predispose to language difficulties, some studies have suggested that the deficient language processing may be due to an underlying cognitive difficulty, for example challenges in processing speed (Marchman et al., 2018). These deficits are not limited to expressive or receptive language use, but are apparent even in neural responses to auditory stimuli, where preterm infants show different responses than their full-term peers (Fellman et al., 2004; Kostilainen et al., 2020).

To support language development in preterm infants, several studies have opted to utilize music or music therapy (Virtala and Partanen, 2018). However, musical interventions may influence infant development in two different ways. First, music has been used in neonatal intensive care units (NICUs) and results show beneficial effects for the preterm infant itself, such as lower heart rate, increased calorie intake, and changes in sleep patterns (Loewy et al., 2013). Second, studies combining singing and kangaroo care have shown reduced levels of infant distress and maternal anxiety (Arnon et al., 2014), which may benefit the preterm infant when the caregiver can provide better parenting. However, while music therapy approaches show promise in supporting neurodevelopment of preterm infants, more evidence is needed (Bieleninik et al., 2016).

Singing uses many low-level auditory features, such as pitch, tempo, and loudness, to emphasize certain parts of the words providing infants with additional cues for speech sound discrimination. Even normal infant-caregiver interaction is highly musical in nature, so additional singing during kangaroo care could facilitate auditory development, resulting in better auditory discrimination skills, and possibly better language outcomes. Consistent with this, singing has been shown to be beneficial in supporting language development in congenitally deaf children after cochlear implantation (Torppa et al., 2014). If singing during kangaroo care would help auditory development or improve auditory discrimination, this should result in differences in neural processing of auditory features between infants born preterm receiving kangaroo care that incorporates singing and those that receive standard kangaroo care. Kostilainen et al. (2021) have already published initial results from our two-center randomized controlled trial (RCT) investigating how kangaroo care that incorporates singing influences auditory processing at term. The results show that kangaroo care that incorporates singing can enhance auditory processing measured with auditory event-related potentials, but the effect may be sex-specific.

The sex differences in the efficacy of singing intervention reported by Kostilainen et al. (2021) are not easy to interpret. Although gender differences are a highly debated topic, some results suggest that there is a small but noticeable difference in language development that females develop slightly faster than males (Wallentin, 2020). However, it is unknown whether this is due to innate or genetic influences, or environment, since there is evidence that parents talk more to female than male children (Leaper et al., 1998). While this issue should be considered, reviews suggest that findings seem inconsistent (Etchell et al., 2018) and sex differences are reported more often in studies with smaller rather than larger number of participants (Wallentin, 2009).

One of the most commonly used approaches to investigate cerebral event-related responses (ERPs from EEG) or event-related fields (ERFs from MEG) is to study Mismatch Negativity (MMN), the brain’s automatic change-detection response originating predominantly from the brain’s auditory cortex (Näätänen et al., 2007). The current model of the MMN posits that when a stimulus violates the auditory system’s expectation of incoming input, the MMN response is generated and is seen as a negative deflection in the frontocentral EEG channels approximately 200 ms from change onset in adults (Winkler, 2007). As the response is not dependent on attention, this method can be used in infants and has even been recorded from sleeping infants (Sambeth et al., 2009). However, in infants, both positive and negative responses are found and thus, the term MMR is used in pediatric populations. While most studies have assessed infant MMR using EEG, a few studies have also been conducted using MEG and the results are highly similar to EEG studies (Sambeth et al., 2006, 2009).

Preterm infants may show smaller or non-existent MMRs in comparison to full-term infants (Kostilainen et al., 2020), or they may show responses of opposite polarity instead, such as a broad positive response in preterm born infants at term and a negative response at 1 year of age (Fellman et al., 2004). MMR amplitude is associated with performance in cognitive tests measuring language skills at 5 years of age, suggesting that studying the MMR could provide useful data in predicting developmental trajectories (Mikkola et al., 2007). However, the MMR is also influenced by maturation and one prevalent view is that the response shifts from positive to negative during early infancy (Leppänen et al., 2004). Indeed, some authors argue that the reduction in MMR amplitude is a beneficial developmental trajectory, as the positive infant MMR may reflect attentional processes (Kushnerenko et al., 2013) propose that the reason why the infant MMR decreases in amplitude in infancy is due to improved inhibitory processes, making it easier to suppress task-unrelated information. This ability to better inhibit processing of task-unrelated information would then no longer cause large positive neural responses due to involuntary attentional shifts similar to those resulting from the P300-response in adults. As result, the MMR amplitude would diminish and eventually shift from positive to negative.

We hypothesized that if singing during kangaroo care would help auditory development or improve auditory discrimination, this should result in differences in MMR amplitude between preterm infants receiving kangaroo care combined with singing and infants only receiving standard kangaroo care. We set out to test this hypothesis in a two-center randomized intervention using similar stimuli paradigms, in which auditory processing was assessed using short pseudowords (“tata” or “tatata”) as stimuli. The pseudowords were adjusted to match the native language of the country where the intervention was conducted. In the primary center, Helsinki University Hospital, preterm infants were assessed with auditory event-related responses in EEG showing that the MMRs were larger in the singing group, especially in females (Kostilainen et al., 2021).

In this study, where infants were recruited in the other center, Karolinska and Sachsska neonatal units, Stockholm, we assessed the responses with MEG. We also recorded MMRs from full-term healthy infants to ensure that they show expected MMRs in our experimental condition.

## Materials and Methods

### Participants

The current data are based on a subsample of the Swedish cohort of the Singing Kangaroo study, in which a total of 43 preterm infants were recruited from Stockholm region hospitals: Karolinska/Danderyd and Sachsska Children’s and Youth Hospital. The following inclusion criteria were used: the infant had been born at GW 32 or earlier, had to be clinically stable, and parents had to be fluent in either Swedish or English. The participants were recruited at the earliest when the infant was 28 GW. Randomization into groups was done by rolling a dice.

Out of the 43 preterm infants, 21 participated in MEG recording (13 in the singing intervention group, 9 female; 8 in the control, and 3 female) at term corrected age, meaning 40 GW (Table 1). Of the 22 infants not participating in the MEG recordings, 8 infants were assessed during development of the MEG paradigm, 9 infants were not assessable since they were still on ventilation support, the parents of one declined the assessment, and the families of four had dropped out of the study. To verify that the experimental paradigm showed expected responses in MEG, 12 full-term healthy infants (8 female) were studied. When eligible families arrived at the delivery hospital, the music therapist (author PH, informed by the head midwife) approached the family and obtained informed written consent to participate in the study. A separate written informed consent was required for the MEG recording after the parents had seen the MEG laboratory and the MEG recording procedure was explained to them.

**TABLE 1:** Background information for the preterm infants participating in the experiment.

	Singing intervention group	Preterm control group	Full-term control group
Participants	13 (9 female)	8 (3 female)	12 (8 female)
GW at birth (weeks + days)	24 + 0–31 + 4 (female: 24 + 0–30 + 4; male: 24 + 0–31 + 4)	27 + 2–32 + 0 (female: 27 + 2–32 + 0; male: 29 + 2–31 + 6)	38 + 2–41 + 5 (female: 38 + 2–41 + 5; male: 39 + 5–41 + 1)
Birth weight (g)	660–1725 (female: 660–1725; male: 700–1580)	870–1716 (female: 870–1716; male: 1054–1625)	3315–4390 (female: 3315–4270; male: 3585–4390)
Number of infants born small for gestational age (SGA)	4 (female: 2; male: 2)	4 (female: 2; male: 2)	0
GW at MEG measurement (weeks + days)	38 + 6–43 + 5 (female: 38 + 6–32 + 1; male: 38 + 6–43 + 5)	39 + 3–43 + 1 (female: 39 + 6–43 + 1; male: 39 + 3–43 + 1)	39 + 3–42 + 3 (female: 39 + 3–42 + 3 male:39 + 5–41 + 1)

For their participation in the MEG experiment, the parents were paid 500 Swedish kronor (approximately 50 USD) and their travel costs were reimbursed. The Singing Kangaroo randomized control trial, including the present study, was granted ethical approval by the Ethics Committee of the Hospital District of Helsinki and Uusimaa, Finland (65/13/03/03/2012). The Swedish part of the study was ethically approved by the Swedish Ethical Review Authority (registry number 2014/1318-31). The intervention was registered in Clinical Trials (ID IRB00003181SK).

### Singing Kangaroo Intervention

The families assigned to the singing intervention group were encouraged by a trained music therapist to sing or hum to their infant during kangaroo care. The intervention was started earliest at GW 28 and lasted for 4 weeks. The music therapist met the parents twice a week during their kangaroo care sessions. The families in the control group received standard kangaroo care and were visited by the music therapist, who only provided support for parenting at a general level. The parents in the control group were not prevented from singing to their infant. To assess the amount of kangaroo care (skin-to-skin contact) and parental singing, the parents in both groups kept a diary and recorded the approximate daily amounts of skin-to-skin contact and singing.

The music therapy sessions for the singing intervention group were approximately 40–45 min each with a focus on inspiring and supporting parents in how they could sing and/or talk in an infant-directed way to their baby. The parents preferred to sing in a lullaby style: a warm and tender voice timbre, slow tempo, repetitive melody, and humming without words in synchronization with the baby’s breathing and movements. The sessions were varied and interactive, tailored to the parent’s needs: singing, humming, toning, or vocalizing together, sharing songs and learning new ones, or supporting the parents in finding their own unique voice (Haslbeck and Hugoson, 2017).

### Magnetoencephalography Stimuli

Two different experimental paradigms were used and always presented in the same order. First, the “tata” experiment, assessing speech sound discrimination, was presented with a multi-feature MMN paradigm (Näätänen et al., 2004; optimum-1 in the original manuscript), in which every other stimulus is a repeating standard sound and every other is one of the deviant sounds, differing from the standard sound in one stimulus feature only. Thus, the probability of the standard tone was 50% and each of the deviant categories had a probability of 10%. The standard sound was the pseudoword “tata,” spoken by a native Swedish speaking female. The tata pseudoword was 300 ms in duration, with the second/ta/syllable starting at 170 ms from pseudoword onset. The gap between the two syllables was approximately 62 ms long and the vowel duration was approximately 82 ms. Five different deviant sound categories were used: the vowel identity deviant (tato), vowel duration deviant (tataa; length increased by 55 to 137 ms, approximate 67% increase), intensity or loudness deviant (two types: second syllable loudness was increased or decreased by 6 dB), pitch increase (two types: second syllable pitch was increased by a semitone or two semitones, approximately 8% or 15%), and pitch decrement (two types: second syllable pitch was decreased by one or two semitones). The change onset between the standard and the deviants was approximately 182 ms from stimulus onset, except for the duration deviant, for which the difference onset was at approximately 264 ms from stimulus onset. For the original version of the tata paradigm, see Partanen et al. (2011). A similar paradigm with Finnish speech sounds was used in a study of infants by Partanen et al. (2013a).

The deviant stimuli in the tata paradigm were created by modifying the standard tata sound in Adobe Audition CS6 5.0. Build 708 (Adobe Systems Inc., California, United States), with the exception of the vowel identity deviant. For the vowel identity deviant, the second syllable from the separately spoken tato pseudoword by the same native female speaker of Swedish was cut and cross-spliced onto the original tata pseudoword and edited in Adobe Audition CS6 so that the consonant and vowel onsets matched the original tata pseudoword and the vowel length was identical in both the standard tata and the deviant tato pseudowords. Finally, the loudness (sound pressure level) of the vowel identity deviant matched to that of the standard by root mean square normalization.

In the tata paradigm, the stimuli in each deviant category were presented a total of 150 times. As some stimulus categories (loudness deviant, pitch increase deviant, pitch decrement deviant) consisted of two stimuli, each of these stimuli were thus presented 75 times each. The tata paradigm started with three standard tata pseudowords, which were removed from further analysis. Stimulus onset asynchrony (SOA) of 1 s was used, with ±25 ms jitter to reduce phase-locked brain activity to regularly repeating stimuli. In total, the tata paradigm lasted approximately 25 min and a total of 1503 stimuli (750 deviants and 753 standards) were presented.

The second experimental paradigm was an oddball paradigm with pure tones. A repeating tone of 1000 Hz served as a standard (probability 80%) and a tone of 1200 Hz served as a deviant. Both tones were 200 ms long with 20 ms rise and fall times. A jitter of ± 25 ms was used and SOA was 800 ms. A total of 120 deviants were presented, and the oddball paradigm lasted a total of 7 min.

The reason for not counterbalancing the experimental paradigms was that during pilot experiments the infants failed to fall asleep during the oddball experiment. Thus, for infant comfort and in order to obtain reliable data, all MEG recordings started with the tata paradigm.

### Magnetoencephalography Procedure

Prior to MEG recording, the parents were encouraged to feed the infant to keep the infant calm during the MEG recording session. First, head position indicator coils (HPI coils) were attached. To avoid discomfort, a flexible cloth cap was fastened to the infant’s head using tape, to which four HPI coils were then positioned and fastened with tape. After HPI coils were attached, the position of the four HPI coils in relation to the cardinal points of the head (nasion, left and right preaurical points) were recorded using an Isotrak 3D-digitizer (Polhemus, Colchester, VT, United States). Two additional electrodes were placed on the infant’s temple to monitor ECG.

Once HPI coils and ECG electrodes were attached, the infant was carried to the shielded room with the MEG device. The dewar was placed in a supine position. Soft cloth was placed on the bed and another thin cloth placed inside the dewar to ensure the comfort of the infant. Inside the dewar, the infant rested on his/her side so that the left side of the head faced the sensors in the occipital part of the MEG helmet. Once the head was placed into the dewar and the infant was calm, the shielded room was closed.

Two persons were present with the infant inside the shielded room. The first was a specialist nurse, whose task was to visually observe the breathing and movement of the infant for signs of medical distress. The second was a researcher, who kept track of the infant behavior during the experiment and using a response pad, pushed a button whenever the infant was moving or otherwise active, thus sending a corresponding signal (trigger) into the MEG recording software. Three different activities were marked: mouth movements (sucking), eye movements, and head movements. A fourth button was available to denote activities that did not fit any of the previously mentioned, but was not used in any of the recordings.

Once the nurse inside the shielded room deemed the infant calm and gave a visual cue via a camera monitoring system to signal that it was permitted to start the MEG experiment, two researchers operating the MEG device outside the shielded room initiated the trial. During the experiments, sounds were played from a non-magnetic loudspeaker in the room (Sound Showers, SSHP60 × 60 W, PanPhonics, Finland). The task of the nurse was to keep the infant still and calm while continuously monitoring the condition of the child, and with parents’ permission, administer glucose solution orally when the infant became fussy. Pacifiers were not used, as excessive mouth movements could contaminate the MEG signal. After the tata paradigm had been successfully completed, the nurse could either signal that the experiment could continue with the oddball paradigm or alternatively ask for a break to feed the infant. If the infant became distressed, the nurse had the option to stop or pause the MEG recording.

### Magnetoencephalography Data Acquisition

The MEG recordings were conducted in a shielded room with an Elekta Neuromag TRIUX whole head MEG system (Elekta Neuromag, Elekta AB, Sweden). The TRIUX 306-channel helmet-shaped MEG device has a total of 102 sensor elements, consisting of two orthogonal planar gradiometers and one magnetometer each. The infant’s head position in the MEG dewar was monitored using the continuous head position indication (cHPI) option in the TRIUX system. A sampling rate of 1000 Hz was used.

### Magnetoencephalography Data Analysis

Typically, the continuous MEG data are first pre-processed using the Spatiotemporal Signal Space Separation method (tSSS) of the MaxFilter software (Elekta Neuromag, Elekta Oy, Helsinki, Finland) to reduce effects arising from external artifacts and to compensate for artifacts arising from head movements. However, the infant head is extremely small in relation to the MEG helmet and a comparison of pilot data analyzed with and without MaxFilter indicated (not reported here) that the ERPs were drastically altered when MaxFilter was used without a visually detectable change in signal-to-noise ratio. Thus, the data were not preprocessed using tSSS.

Data analysis was performed with Brainstorm (Tadel et al., 2011), which is documented and freely available for download online under the GNU general public license^1^ and Matlab R2018b (The MathWorks Inc., Massachusetts, United States). The continuous MEG data were first filtered from 0.1 to 45 Hz to remove high-frequency cHPI artifacts. After the initial filtering, the data were visually inspected. When any artifact was observed in the data co-occurring with triggers signaled by the researcher, who had observed the infant during the MEG recording, the artefactual data segments were rejected from further analysis. Then, heartbeats were detected from ECG, or if the ECG data was poor, from MEG channels with the most prominent heartbeat. Artifacts arising from heartbeats were corrected for using Brainstorm’s signal-space projection (SSP) algorithm, separately for gradiometers and magnetometers. To remove high-frequency activity and reduce low-frequency drift, the data were finally filtered from 1 to 30 Hz. For further analysis, data were divided into 900 ms epochs, starting from 100 ms prior to stimulus onset and ending 800 ms after stimulus onset. All accepted epochs were averaged together, separately for each infant and stimulus category (standard tata, vowel identity, vowel duration, vowel loudness, small pitch change, and large pitch change for tata; standard and deviant for oddball).

As the head position in the MEG dewar could differ between infants, we strove to select the sensors in a systematic manner. As magnetometers are more susceptible to noise and artifacts, all magnetometers were excluded from the analyses. Only data from gradiometers in the occipital region of the MEG helmet were used (48 gradiometers; 24 gradiometer pairs), as the head was resting on them and were most likely to show signals originating from the brain. Norms of gradiometer pairs were calculated to obtain a direction-independent signal from each sensor. Subsequently, for each infant and separately for the two paradigms, the gradiometer pair selection was conducted according to the following steps. First, on the basis of previous literature, we determined that the expected time window in which the MMR should appear was between 200 and 500 ms from change onset (Kostilainen et al., 2020). Second, deviant-minus-standard waveforms (MMR waveforms) were calculated, separately for each paradigm (tata, oddball) and deviant category. Third, the response magnitude in the MMR waveform in the time window of interest (400–700 ms from stimulus onset for tata and 200–500 ms for oddball, corresponding to approximately 200–500 ms from change onset for both paradigms) was calculated for each gradiometer pair. Fourth, the response magnitude in the MMR waveform outside the time window of interest was calculated for each gradiometer pair.

To select the gradiometer pairs for further analysis, the gradiometer pair had to fulfill two conditions. First, the MMR response magnitude in the time window of interest should be greater than the average response magnitude of MMR waveform outside the time window of interest over all the gradiometer pairs. This criterion aimed to select channels that had a response in the time window of interest. Second, the response magnitude outside the time window of interest should be smaller than 3 fT in order to avoid including gradiometer pairs that have high-amplitude noise across the whole epoch. The limit of 3 fT was chosen by visual inspection of the data and subjective assessment of the amplitude of the background noise observed across participants. For tata, the gradiometer pair had to satisfy the two aforementioned conditions for three of the five deviant types to be included for further analysis to avoid the inclusion of channels that randomly show a response for one or two deviant types. If this method did not result in any accepted gradiometer pairs, a second pass was conducted in order not to reject infants with small responses, but only for those infants whose data failed to satisfy the first criteria. In the second pass, gradiometer pairs were accepted if the response magnitude outside the time window of interest was smaller than 3 fT regardless of the response magnitude in the time window of interest.

One infant in the singing intervention group was accepted into further analysis after the second pass and zero in the control group. One infant in the singing group (male) was left with no accepted gradiometer pairs and was excluded. Infants in the singing intervention group had an average of 5.92 accepted gradiometer pairs (range 1–9) for tata and 5.46 for oddball (range 1–17). The control group had an average of 4 (range 2–8) accepted gradiometer pairs for tata and 6.25 (range 3–16) for oddball.

In the full-term group, three infants were accepted into further analysis after the second pass. The group had 5.75 (range 1–14) accepted gradiometer pairs for tata and 5.83 (range 3–14) for oddball. The number of accepted gradiometer pairs did not differ between groups (*p* = 0.893 for oddball and *p* = 0.486 for tata). To obtain a single MMR waveform for each individual, responses from all accepted gradiometer pairs were averaged together.

### Statistical Analyses

Statistical analyses were conducted in SPSS 25 (IBM Corporation, NY, United States). Using a similar approach as Fellman et al. (2004), the MMR waveform was divided into 100 ms time windows (400–500, 500–600, 600–700 ms for tata, and 200–300, 300–400, 400–500 ms for oddball) and mean MMR amplitude was calculated in each time window, separately for each infant and deviant category.

The statistical significances of the MMRs were tested using one-tailed *t*-tests, comparing the magnitude of the MMR to zero using a one-tailed *t*-test. One tailed *t*-tests were used as the responses of the gradiometer pairs are calculated as a square root of a sum of squares. Thus, they are larger than zero by definition.

MMR differences between infants born preterm and full-term were assessed using rm-ANOVA, separately for tata and oddball paradigms. Used between-groups factors were Sex (male and female) and Group (full-term, singing intervention, and control) while within-group factors were Time Window (3) and Deviant Category (5). The Deviant Category factor was only used for the tata paradigm as it is redundant for the oddball paradigm which included only one deviant type.

Similar and directly comparable analyses on the efficacy of the intervention on MMR amplitudes to those conducted in the study of Kostilainen et al. (2021) were performed. We conducted an rm-ANOVA with Sex (male and female) and Group (singing intervention and control) as between-groups factors and Time Window (3) and Deviant Category (5; this factor was used for tata only as only one deviant category was used in oddball) as within-group factors. We also included the amount of singing in hours as a covariate, but not time in skin-to-skin contact. Since the total amount of singing and total amount of time spent in skin-to-skin contact were strongly correlated (*r* = 0.452, ρ = 0.288), due to multicollinearity issues, only singing in hours over the 4-week intervention period was used as a covariate. One infant’s diary on skin-to-skin contact and singing was missing and this infant was removed from this analysis (female, singing intervention group).

Any main effects of factor Time Window were ignored in all analyses. When sphericity was violated, the Greenhouse-Geisser correction was used as appropriate (uncorrected degrees of freedom are reported). Effect sizes are reported as partial eta-squared (η^2^).

As our sample size is very small, linear mixed models (LMM) were used to verify the rm-ANOVA since this method is less sensitive to errors arising from small sample sizes. LMM analyses were conducted using R (R Core Team, 2021) and the lme4 package (Bates et al., 2014). To facilitate the comparison of results between LMM and rm-ANOVA, variables were not standardized. Only data from singing intervention and control groups were used. For LMM, average MMRs across all time windows were used. The variables total singing time in hours, Sex, and Group were used, and interactions of Sex and Group, singing time and Sex, and singing time and Group were entered into the LMM models. Females and the singing intervention group were used as reference group for easier interpretation of the results, as larger MMRs were expected for the singing intervention group and females (Kostilainen et al., 2021). Estimates and standard errors are reported.

## Results

The amount of skin-to-skin contact (singing intervention: 195.75 h, control group: 133.96 h, *p* = 0.058) or singing time (singing intervention: 33.67 h, control group: 28.21 h, *p* = 0.658) did not significantly differ between the groups. When assessing skin-to-skin contact separately for both parents, no differences were found for skin-to-skin contact (singing intervention: 113.11 h, control group: 110.76 h, *p* = 0.924) or singing time (singing intervention: 21.27 h, control group: 25.82 h, *p* = 0.634) with mothers. In contrast, fathers in the singing intervention group had more skin-to-skin contact with their preterm infants (singing intervention: 82.63 h, control group: 23.20 h, *p* = 0.002) and sang to them more (singing intervention: 12.40 h, control group: 2.39 h, *p* = 0.023).

### Magnetoencephalography Results

**FIGURE 1:**
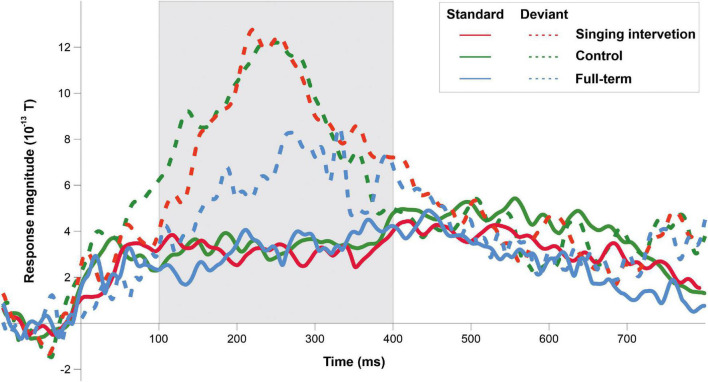
ERP responses to the standard tones (solid lines) and deviant tones (dashed lines) for all groups in the oddball paradigm. Grayed area denotes the predefined MMR time window.

**FIGURE 2:**
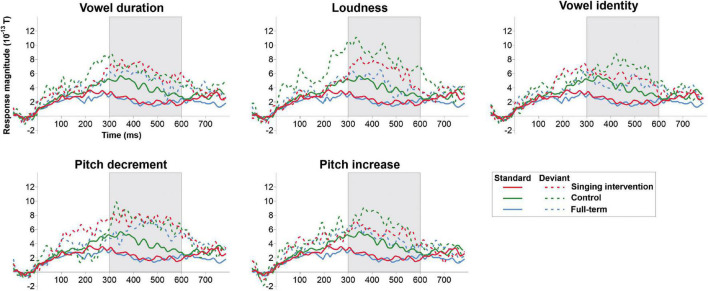
ERP responses to the standard tata (solid lines) and responses to all deviant types (dashed lines) for all the groups in the tata paradigm. Grayed area denotes the predefined MMR time window.

**FIGURE 3:**
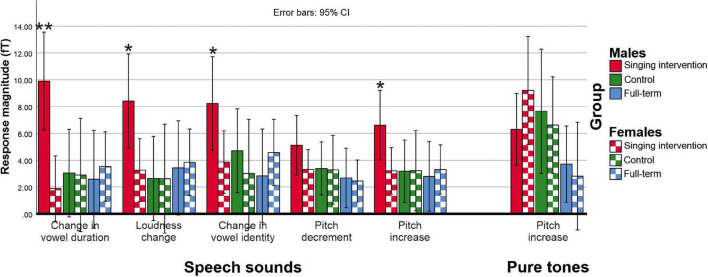
Deviant minus standard MMR responses for each deviant category for both speech sounds (tata paradigm) and pure tones (oddball paradigm). Asterisks (*) denote deviant categories in which males in the singing group showed larger responses than females in the singing group (*p < 0.05; **p < 0.01).

ERF waveforms for the standard and deviant tones for the oddball and tata paradigms are depicted in Figure 1 and Figure 2, respectively. MMRs for all the stimuli across the three time windows, separately for both sexes and for both paradigms, are shown in Figure 3.

Full-term infants elicited statistically significant MMRs to all deviant types in both tata and oddball conditions (*p* < 0.008 for all comparisons). Also, the MMRs in the singing intervention group (*p* < 0.024 for all comparisons) and control group (*p* < 0.038) were statistically significantly different from zero.

In the oddball paradigm, an effect of Group (full-term, singing intervention, control) was statistically significant [*F*(2,27) = 4.019, *p* = 0.030, η^2^ = 0.229], and *post hoc* tests indicated that infants in the singing intervention group showed larger responses than full-term infants (*p* = 0.023). The difference between full-term and control groups was nearly significant (*p* = 0.055). No overall effect of Sex was found in the oddball paradigm [*F*(1,27) = 0.051, *p* = 0.823, η^2^ = 0.002], and the interaction between Group and Sex was not statistically significant [*F*(2,27) = 0.822, *p* = 0.450, η^2^ = 0.057].

In the tata paradigm, no main effect of Group was found [*F*(2,27) = 2.332, *p* = 0.116, η^2^ = 0.147]. No main effect of Sex was found in the tata paradigm [*F*(1,27) = 2.042, *p* = 0.164, η^2^ = 0.070]. However, a three-way interaction of Group, Sex, and Stimulus was found [*F*(8,50) = 3.120, *p* = 0.039, η^2^ = 0.136]. On the basis of *post hoc* tests, the responses of males in the singing intervention group were larger than responses of females in the full-term (*p* = 0.016) or the control groups (*p* = 0.023), and also larger than responses of females in the singing intervention group (*p* = 0.008). Further investigating the three-way interaction of Group, Sex, and Stimulus, we found that males in the singing intervention group showed larger responses than females in the singing intervention group for all stimulus types except for pitch decrements (vowel duration: *p* = 0.001; loudness change: *p* = 0.019; vowel identity change: *p* = 0.042, pitch decrement: *p* = 0.170; pitch increase: *p* = 0.033).

In analyses testing the efficacy of the intervention including the total singing time as a covariate, it became apparent that singing had a different effect on MMRs in tata paradigm in singing intervention and control groups, and in males and females. First, we found an interaction between Sex and total singing time [*F*(1,16) = 7.162, *p* = 0.017, η^2^ = 0.309] and Group and total singing time [*F*(1,16) = 5.674, *p* = 0.030, η^2^ = 0.262] in the tata paradigm. Based on parameter estimates and their regression coefficients, the effect resulted from two factors. First, in the singing intervention group, the regression coefficients seemed larger than in the control group, suggesting that the effect of singing on MMR was larger in the singing intervention group than in the preterm control group. Second, the regression coefficients appeared smaller for females than for males regardless of the group, indicating that the enhancement of the MMR due to singing were larger for males than for females. No intervention effects were found in the oddball paradigm.

In LMM analyses used to verify the rm-ANOVA results, regression coefficients were mostly in line with rm-ANOVA results, as seen in Table 2. The amount of singing was associated with larger responses in males than females and larger responses in the singing intervention than control group in the tata paradigm. The effect was statistically significant only for vowel identity and loudness changes. However, the effect was small.

**TABLE 2:** Linear mixed model results.

	Pure tones	Speech sounds				
	Pitch increase	Change in vowel duration	Loudness change	Change in vowel identity	Pitch decrement	Pitch increase
Amount of singing	0.054 (0.070)	−0.034 (0.047)	−0.065 (0.083)	0.028 (0.037)	−0.049 (0.079)	−0.037 (0.054)
Sex	8.798 (5.380)	−0.842 (3.587)	−8.689 (6.397)	−5.730 (3.034)	−6.952 (5.990)	−1.152 (4.072)
Singing vs preterm control	−0.249 (3.942)	−1.302 (2.689)	7.873 (4.688)	6.759* (2.091)	2.324 (4.930)	1.994 (3.183)
Sex * Group	−6.902 (4.209)	2.550 (2.902)	2.140 (5.132)	1.680 (2.290)	1.834 (4.847)	−1.071 (3.306)
Sex * Amount of singing	−0.191 (0.171)	0.093 (0.115)	0.462* (0.204)	0.352*** (0.091)	0.346 (0.193)	0.121 (0.132)
Group * Amount of singing	0.144 (0.172)	−0.073 (0.116)	−0.467* (0.205)	−0.396*** (0.091)	−0.285 (0.193)	−0.128 (0.132)

*p < 0.05 and ***p < 0.001. Asterisks denote statistical significance.

## Discussion

The main findings of the present study were that neural responses to changes in speech sounds were enhanced in the singing intervention group in comparison to the control group when controlling for total singing time. Furthermore, our data also suggest that some sex differences seemed to appear when incorporating singing into standard kangaroo care.

Our results are consistent with the findings of Kostilainen et al. (2021) and illustrate that parental singing during kangaroo care may influence the discrimination of changes in speech sounds in preterm infants. However, our results also seem to imply that how parental singing is conducted may affect the results. When controlling for the total amount of singing, we found that the singing intervention group showed larger MMRs than the control group. As parents in both intervention and control groups sang to their infants, this could imply that exposure to singing may not benefit the infant in itself. Instead the effect might appear when singing is conducted in a manner that is most beneficial to the parent-infant interaction. As the parental singing was facilitated by a trained music therapist in the singing intervention group, it would be plausible that the music therapist could guide and inspire the parents to use music in a manner that would be developmentally useful. This could then result in beneficial effects seen at term age.

The finding that there were no differences in total singing time between the singing intervention and control groups also supports the interpretation that the amount of singing during kangaroo care during the 4 weeks of intervention in itself may not be beneficial for influencing the MMR responses. As the amount of singing was highly correlated with the amount of time spent in skin-to-skin contact, it suggests that parents tend to sing or hum to their preterm children even during normal kangaroo care. Although the possibility of a random effect cannot be ruled out due to small sample size in our study, similar findings were observed in the analyses of the Finnish cohort of the Singing Kangaroo study (Kostilainen et al., 2021), although in their study the total amount of singing was positively correlated with MMR amplitudes while the daily amount of singing was not. Taken together, this could be interpreted that possibly other aspects of the music therapy than the amount of daily singing in itself may drive the enhancement seen in MMRs. It seems possible that our tentative results could be due to improved caregiver sensitivity and attachment arising from interactive activities conducted with the music therapist. This interpretation is in line with Virtala and Partanen (2018), who propose that musical interventions may have the largest effects when it is interactive and fosters positive interaction between the caregiver and the infant.

However, the larger MMRs might not necessarily indicate strictly beneficial effects. For example, Kushnerenko et al. (2013) argue that the infant MMR shifts in infancy from positive to negative, and interpret this shift as a typical development where the infant learns to inhibit attentional shift to non-relevant stimuli. Kushnerenko et al. (2013) posit that this would result in reduction of the P300 response amplitude, a positive deflection, and allow the negatively displaced MMN to be observed in similar latencies. Furthermore, we did not find differences in MMR amplitude between the control group and the full-term group in the oddball paradigm even though the MMRs of the singing intervention group were larger than those of the full-term group. If the MMR of the full-term infants represents a baseline for typical development and the preterm control group shows similar MMRs, can larger MMRs in the singing intervention group be considered beneficial for development? Whether greater MMR response amplitude has long-term beneficial effects for cognition will be assessed in the follow-up, when the infants return for cognitive testing at 2 years of age. By combining the present data with the data from this same two-center RCT study, reported by Kostilainen et al. (2021), the sample size might be large enough to assess any possible long-term effects of the larger MMRs in the singing intervention group.

We observed a sex difference, namely that males in the singing intervention group had larger MMRs than females. Males and females may have somewhat different developmental trajectories in infancy, possibly explaining the different reactivity to the singing intervention in this study. This is in line with the reported sex differences in MMRs to speech sounds in infancy in two studies (Friederici et al., 2008; Mueller et al., 2012). In addition, EEG coherence may differ between the sexes (Hanlon et al., 1999). Although sex differences are also found in newborn behavior, there are more similarities than differences between the sexes (Boatella-Costa et al., 2007). Language development, and thus sensitivity to speech sound changes, may be more rapid in females than in males early on, as indicated by the study of Bouchard et al. (2009), where Canadian 8–30 month old females had better language competencies than males. It has been suggested that the effect might arise from females’ better ability to use their long-term memory representations in word learning than males (Kaushanskaya et al., 2013). However, our results showing enhanced MMRs in males are inconsistent with this view. It is very likely that the observed sex difference is a random finding due to the small sample (Wallentin, 2009) as it is the opposite of what was found in the Finnish study (Kostilainen et al., 2021).

Another aspect of the present study is that we found larger MMRs in infants born preterm than in full-term infants. Infants born preterm have repeatedly been shown to exhibit different MMRs to non-speech sounds in comparison to their full-term born peers (e.g., Fellman et al., 2004; Paquette et al., 2015; Kostilainen et al., 2020). However, the reported responses in preterm infants have been delayed or absent, while the present study indicated greater response magnitudes in preterm-born infants. Although the reason for different result in the present experiment is not clear, several interpretations are possible. First, it is plausible that neural sources accounting for the MMR differ between the current and previous experiments. As the current study was conducted using MEG and the earlier studies were performed using EEG, MMR components radial (directly toward or away from) to the scalp are not seen in the present experiment as the MEG is not sensitive to those. In addition, as only one hemisphere was recorded and the infant MMR is hypothesized to arise from several neural sources (Piazza et al., 2016), our results may only show effects on preterm birth on sources near the left temporal region. Second, the reduced or absent MMRs studies might be due to maturation. As the MMR seems to change from positive to negative during development (Leppänen et al., 2004), averaging across infants who show MMRs of different polarity may result in either very small or absent responses. Consistent with this, some studies have reported infants with both negative and positive MMRs (Partanen et al., 2013b). However this effect might is cannot be seen in MEG analyses using norms of gradiometer pairs, as MMR amplitude is measured using absolute values. This could potentially explain why our results differ from those in previous studies.

For multiple reasons, our results need to be interpreted with care. First, sample sizes were small and conducting analyses using linear mixed models revealed that our observed effects were highly likely to be small. However, the Singing Kangaroo project consists of two separate cohorts, where data collection, intervention, and analysis are conducted independently of each other, and thus comparison of findings between the two cohorts will help to assess the veracity of the present findings. Both the Finnish (Kostilainen et al., 2021) and the Swedish cohorts are similar in that parents were assisted in singing with their preterm infants in kangaroo care settings. Furthermore, similar experimental paradigms have been used and final outcome measures have been chosen. Comparisons of EEG and MEG results need to be performed with care, and the present MEG results need to be interpreted carefully. While infant EEG studies are common, use of MEG to study term infants is rare and technically challenging and is limited to recording data from one hemisphere only.

In addition, MEG recordings prior to the intervention were not possible due to neonatal morbidities at 26–30 GW. Thereby, it was not possible to assess changes in MMRs due to the singing intervention. Whether larger MMRs found in the singing group are associated with developmentally beneficial effects is unclear, but we aim to assess the cognition of the studied infants at 2 years of age.

To summarize, incorporating music therapy approaches to standard skin-to-skin care for preterm infants might result in enhanced MMRs to changes in speech sounds. As the effect may not arise specifically from singing itself, it would seem plausible that music therapy approaches may benefit the infant indirectly, via positive parenting, improved parental self-esteem and improvement of caregiver sensitivity. As a result, the attachment between the infant and the caregivers may improve, and have broader and longer-lasting benefits on child development than interventions aimed at a very specific target.

## Data Availability Statement

The raw data supporting the conclusions of this article will be made available by the authors, without undue reservation.

## Ethics Statement

The Singing Kangaroo randomized control trial, including the present study, was reviewed and granted ethical approval by the Ethics Committee of the Hospital District of Helsinki and Uusimaa. (Clinical Trials ID IRB00003181SK). The now reported subpart of the study was also approved by the Swedish Ethical Review Authority (registry number 2014/1318-31). Written informed consent to participate in this study was provided by the participants’ legal guardian/next of kin.

## Author Contributions

PH, MH, VF, and UÅ planned the study design. PH and UÅ recruited the families. PH guided parents to conduct the intervention and stored the diary data. UÅ was the corresponding physician in the study and led the research group. VF was responsible for the whole Singing Kangaroo project, including the Swedish and Finnish cohorts. EP, GM, and PH conducted the MEG recordings. EP and GM analyzed the data. EP conducted the analyses and wrote the initial version of the manuscript. All authors commented and reviewed the manuscript draft.

## Conflict of Interest

The authors declare that the research was conducted in the absence of any commercial or financial relationships that could be construed as a potential conflict of interest.

## Publisher’s Note

All claims expressed in this article are solely those of the authors and do not necessarily represent those of their affiliated organizations, or those of the publisher, the editors and the reviewers. Any product that may be evaluated in this article, or claim that may be made by its manufacturer, is not guaranteed or endorsed by the publisher.

## Footnotes

1
http://neuroimage.usc.edu/brainstorm
